# Variation in perceived health across gender, working status, educational level, and regional health care expenditure in Spain (2014–2017)

**DOI:** 10.1371/journal.pone.0269613

**Published:** 2023-07-14

**Authors:** Amanda Godoy-Bermúdez, Araceli Rojo-Gallego-Burin, Luisa Delgado-Márquez, José J. Martín-Martín, M. Teresa Sánchez-Martínez, M. Puerto López del Amo-González

**Affiliations:** 1 Applied Economics Department, University of Granada, Granada, Spain; 2 Management Department, University of Granada, Granada, Spain; ISEG Lisbon School of Economics and Management, PORTUGAL

## Abstract

A gender perspective was used to analyze whether and how education, unemployment, and per capita public health expenditure were associated with perceived health among the Spanish population between the years 2014 and 2017. Using multilevel methodologies (looking at year, individual, and region) and linear and logistic specifications, we analyzed longitudinal microdata files from the Survey on Living Conditions. The results suggest that women with lower educational levels tend to report worse health than their more educated counterparts. On the other hand, women’s bad health was not associated with unemployment, unlike men’s. Regional per capita public health expenditure was not associated with perceived health in either men or women.

## Introduction

A conceptual framework concerned with the social determinants of health postulates that financial and social environments are strong determining factors of the health of individuals. Therefore, higher income, educational attainment levels, and being employed are associated with improved health [1–4]. In that frame of reference, gender appears as a particularly influential axis of inequality [5, 6].

Biological differences between sexes are insufficient to explain diverging health trends, which have been proven to be preventable and avoidable [7]. Socially and culturally constructed gender norms determine the roles and opportunities afforded to all individuals and arise as strong structural determinants of health, with major yet different implications for women and men [5, 8]. Such differences become problematic when they give rise to inequality and discrimination, which are particularly detrimental to women. Gender power dynamics, which are among the main causes of gender inequality, are also some of the strongest social determinants of health, with a clear effect on how people are born, grow up, live, work, get old, and finally die [9].

The main goal of the present work is to analyze, from a gender perspective, the association between perceived health and education and unemployment. Additionally, this study examines the extent to which regional public health expenditure is associated with the perceived health of men and women in a given region.

For the most part, the relevant literature consists of partial analyses of the relation between health and certain social and economic variables, such as the role of unemployment or poverty on health [10–16]. In the present study we consider that the link between educational level, working status, and individual health is a key element in the analysis of general socio-economic status. Additionally, we aim to expand on the existing knowledge regarding the gender-based health gap as it relates to levels of educational attainment and working status. The literature on these specific topics is highly contradictory, and precious few studies look into both aspects simultaneously. Our research focuses on analyzing the association between educational level, working status, and perceived health, and how such association differs for men and women. Along these lines we have also identified specific public policies with the potential to reduce the gender gaps identified over the course of our analysis.

Educational level, as the literature attests, is a major social determinant of health. Most analyses confirm that with higher educational attainment comes better health, but also increased longevity in good health. This is made even more apparent when comparisons are drawn with individuals with low levels of education [17–19]. However, the specifics of how the benefits of education are unevenly distributed among women and men have not been sufficiently explained, and the conclusions attained are far from unanimous. Given that women are generally at a disadvantage as far as the distribution of socio-economic resources such as power, authority, earnings, household income, and general wealth is concerned, their health and survival might be more education-dependent than it is for their male counterparts.

This study examines whether the beneficial association of education with health is particularly strong for women, and whether education by itself may suffice to overcome gender differences in perceived health.

Previous research has concluded that women with low educational levels have particularly low self-perceived health compared with similarly educated men after controlling for other characteristics [18–21].

A recent analysis carried out in Spain among an adult population established that, for women, the association between low educational level and poor self-perceived health was particularly strong [22]. According to several cross-sectional studies, the correlation between inequality and having received a lower level of education becomes more marked in the case of women [23, 24]. Thus, women of a low educational attainment level are doubly affected: first, they endure worsened medically-diagnosed health, and therefore perceive their own health as poorer than do more highly educated women; secondly, they experience decreased psycho-social support and are expected to receive less care for the same medical conditions, which in turn increases the perception of their own health status as poor [24]. The theory of resource substitution, according to which the absence of one or several socio-economic resources may be offset by drawing more intensely from the existing ones, predicts that the benefits of education on health and survival are larger for women, with physical deterioration being more intensely reduced for women than for men as their educational level increases. Thus, the gender gap in physical status essentially vanishes as individuals reach college levels of education [25]. The absence of alternative resources makes women’s welfare particularly dependent on their education and training. Thus, poorly educated women may suffer from more and worse health problems because they have less resources to draw from [25–28]. In that regard, it must be noted that the physical condition of women deteriorates less than that of men as levels of educational attainment increase. Therefore, the gender gap in physical deterioration vanishes as women reach a college education level. This hypothesis is supported by data from a 1995 survey among adults in the U.S., with follow-ups in 1998 and 2001 [25].

However, an analysis of the active population in Spain failed to find gender disparities concerning the association of education with health, although it showed that women’s health is poorer among less educated individuals, mainly due to job insecurity and the specific characteristics of households [17].

Some research on psychological welfare pointing at self-perceived health as one of its main correlates [29] suggests that education is more beneficial to women than men [25, 30]. Concretely, a study on subjective wellbeing carried out in South Korea with individual panel data from the period 2017–2020 confirmed the existence of a positive relation between educational attainment level and the perception of welfare, and pointed at clear gender differences in the magnitude of this effect. In that case, the positive impact of higher education was stronger among women [31]. Concerning this matter, the literature suggests several possible rationales for the link between education and welfare. First, education tends to boost the self-esteem of women more than it does that of men [25, 32]. This may be due to the fact that, lacking other resources, women are particularly dependent on their own education to achieve certain levels of welfare. Regardless of the underlying causes of such gender differences, these findings suggest that there exists a potential for women’s education to improve their wellbeing, particularly among older individuals who may have experienced unequal opportunities in the access to education over the last decades [33]. In contrast, the theory of resource multiplication implies precisely the opposite, that education should be more beneficial to men than women as they are able to derive greater rewards from it in the labor market, such as increased authority and income [25, 26].

The critical link between women’s education and health is emphasized even more by evidence concerning the discrimination faced by women who attempt to access the labor market, and by the existing gender pay gap, which has increased as a result of the latest financial recession [34].

Working status, and in particular the employed-unemployed dichotomy, has traditionally played a central role in the analysis of social inequalities concerning health. The intensity and magnitude of the Great Financial Crisis had a profound effect on the Spanish labor market, and the resulting landscape demands that we explore new perspectives and take a closer look at the experience of unemployment and how it interacts differently with the health of women and men [35]. Previous analyses on the association between unemployment and health have revealed, in most cases, a negative relationship [19, 36, 37]. However, there is no consensus in the literature concerning the specifics of how the health effects of unemployment differ between men and women. While Norström et al. (2014) did speak of the effect of unemployment on health, they did not find it to be systematically linked to gender [38]. Meanwhile, Drydakis (2015) found that, during the intense depression caused by the financial crisis in Greece, the detrimental association between unemployment and health was stronger for women than for men [39]. In contrast with Drydakis (2015), Antonakakis and Collins (2014) reached a different set of conclusions in their analysis of the Greek situation, as they found that the financial recession, unemployment and its austerity measures were more starkly associated with male than female suicide rates [40]. Other studies have similarly suggested that austerity, layoffs, and unemployment have had a stronger association with the mental health of men than women [41–43]. Along such lines, Calzón et al. (2017) found evidence of unemployment as a risk factor for bad self-rated health among men, but not women [20]. As for France, Ronchetti and Terriau (2019) did not find a negative link between long-term unemployment and self-perceived health for men nor for women [44]. Buffel et al. (2015), applying a multilevel framework to data extracted from the European Social Survey (2006 and 2012 rounds), looked into the relation between unemployment and mental health across 20 European countries, and found men to be more likely to undergo depression than women [45]. In light of these studies, it becomes necessary to analyze how periods of economic recovery may modify how unemployment interacts differently with the health of women and men, while keeping in mind the singularities of the Spanish economy, characterized by one of the highest unemployment rates in the European Union and a largely unstable and insecure labor market in which women unemployment rate is higher.

At the regional level, we carried out an analysis of regional per capita health expenditure and perceived health, as such expenditure differs greatly across regions. The Public Health Expenditure Statistical Report published by the Spanish Ministry of Health (*Estadística de Gasto Sanitario Público*, 2022), reveals a 48% gap between the highest spending region (Basque Country) and the lowest (Andalusia). The differences are significant: even accounting for the Basque Country’s disparate legislative and administrative framework, the differences amount to 38%. Among low-income countries, higher health expenditure seems to be linked to significant improvements in health status, which suggests that public policy may make a big difference in this regard [46, 47]. However, among high-income countries, additional health expenditure increases appear to be largely unrelated to health status improvements, which implies that increases in expenditure alone do not suffice to improve health to a significant degree [46–48]. Other studies, such as that carried out by Heijink et al. (2013) for 14 Western countries, did find a statistically significant link between health expenditure and avoidable mortality [49]. Nixon and Ulmann (2006) examined the relation between health care expenditure and health outcomes (average life expectancy at birth and infant mortality rate). They concluded that, although health expenditure has contributed significantly to ameliorate infant mortality, it is only associated with marginal improvements to life expectancy in EU countries during the period under analysis [50]. Some researchers have been able to identify that women’s educational attainment levels, technological improvements, per capita income, unequal distributions of income, and certain cultural differences are behind some of the strongest improvements in health outcomes, far beyond increases in health expenditure [51, 52].

To summarize, in Spain as well as internationally, the available evidence concerning the link between education, working status, gender, and perceived health is far from conclusive or unanimous. The present study takes a wide longitudinal database to explore how educational attainment level and working status affect health-related gender inequalities. Additionally, this work analyses how the differences in public health expenditure of each region relate to the perceived health status of their citizens.

## Materials and methods

### Scope of the study

A database was built using the microdata files from the Survey on Living Conditions (2014–2017), comprising 17,027 individuals aged between 16 and 60, with 41,111 observations [54]. By gender, 8,603 women with 20,856 observations and 8,424 men with 20,255 observations were analyzed. Per capita public health expenditure information was extracted from the Public Health Expenditure Statistical Report published by the Spanish Ministry of Health *(Estadística de Gasto Sanitario Público)*.

### Data analysis

We have estimated longitudinal multilevel models (three levels) to better fit the chronological and hierarchical structure of data. First, the whole database was considered, and then we independently estimated a variety of models for women and men. This approach allowed us to test the robustness of our results against several specifications of the dependent variable, widen the range of comparability with other published studies, and analyze the asymmetries that gender may bring to the association between perceived health, educational attainment level, and working status. The first level was the year, the second the individual, and the third level was the region. Analytically for the linear model:

$$y_{ijk}=\beta_{0}+\sum_{h=1}^{H}\beta_{h}x_{hijk}+\sum_{m=1}^{M}\alpha_{m}x_{mik}+e_{ijk}+u_{jk}+c_{k}$$ (1)

where *y*_*ijk*_ is the dependent variable (perceived health), taking values 1–5, for year *i*, being i = 2014, …, 2017; the individual *j*, being *j* = 1,…,17,027; and region *k*, being *k* = 1,…,17. *β*_0_ represents the independent term; *x*_*hijk*_ are the individual explanatory variables; *j* and *β*_*h*_ their associated coefficients, with *h* = 1,…,*H*; *x*_*mik*_ are the explanatory variables at the ecological level and *k* and *α*_*m*_ their associated coefficients, with *m* = 1,…,*M*. The error term divides the unexplained part in three, one for each hierarchical level: *c*_*k*_ at the ecological level, *u*_*jk*_ for the individual, and *e*_*ijk*_ for each year.

Perceived health, the dependent variable in the logit multilevel model, initially comprised five potential answers. These were collapsed into either good or bad perceived health, as this does not substantially alter the relation between socio-economic characteristics and health [53] while increasing comparability with previous studies. Dependent variable Y_ijk_, for year i follows a binomial distribution Y_ijk_ ~ Binomial (1,π_ijk_) with Y variance conditioned by π, Var(Y_ijk_ | π_ijk_) = (1-π_ijk_), where π_ijk_ is the likelihood of exhibiting the feature of interest for year i, being i = 2014, …, 2017; individual j, with j = 1,…, 17,027; and region k, with k = 1,…, 17. Analytically:

$$logit\left(y_{ijk}\right)=\beta_{0}+\sum_{h=1}^{H}\beta_{h}X_{hijk}+\sum_{m=1}^{M}\alpha_{m}Z_{mik}+v_{0k}+\mu_{0jk}+\epsilon_{ijk}$$ (2)

where *β*_*0*_ represents the independent term; *X*_*ijk*_ are explanatory variables at the individual level *j*, and *β*_*h*_ their associated coefficients; *Z*_*ik*_ are the explanatory variables at the regional level *k*, *and* α_m_ their associated coefficients. The error term divides the unexplained part of the dependent variable into three terms, one for each hierarchical level. It is assumed that the components of variance average zero and have constant variance. Thus, the likelihood of individual *j* in year *i* and region *k* exhibiting a given feature of interest is:

$$\pi_{ijk}={\left(1+\text{exp}\left(-X_{ijk}\beta -Z_{{}^{ik}}\alpha \right)\right)}^{-1}$$ (3)

Since the equation in the logistic multilevel regression model represents the likelihood of exhibiting the feature of interest, the exponential of the model’s parameters may be interpreted as an odds ratio.

Multilevel models have been proven to be a good fit for hierarchical structures that include several levels of information, and in which individuals share certain features by virtue of belonging to the same higher level (the region). They are also a good choice when repeated measures exist, given that they allow for variance to be estimated at each level. Multilevel models thus avoid both the ecological fallacy (in which aggregated data are interpreted at the individual level) and the atomistic fallacy (in which individual data are interpreted at the aggregated level) [54]. The software employed to analyze the data was Stata 15. The sintax for the analysis is available in Annex 1 in S1 File.

The dependent variable, perceived health, takes values 1–5, with 1 for very good health and 5 for very bad health. Perceived health is a proxy variable for morbi-mortality, commonly employed in health and living conditions surveys. It is one of the indicators of choice used to monitor gender inequalities and their determinants in matters of health [55–57].

$$logit\left(y_{ijk}\right)=\beta_{0}+\sum_{h=1}^{H}\beta_{h}X_{hijk}+\sum_{m=1}^{M}\alpha_{m}Z_{mik}+v_{0k}+\mu_{0jk}+\epsilon_{ijk}$$ (4)

$$\pi_{ijk}={\left(1+\text{exp}\left(-X_{ijk}\beta -Z_{{}^{ik}}\alpha \right)\right)}^{-1}$$ (5)

Individually, the variables of interest are educational attainment level and working status.

Educational level is defined as the amount of formal instruction successfully completed, and is divided into primary, secondary, and college level. Working status has been characterized using six categories: employed, unemployed, student, homemaker, retired, and other inactive status.

Age and chronic illness (dichotomous variable) were used as control variables. This allowed us to limit the endogeneity problem in analyzing unemployment and education as they relate to perceived health. By considering chronic illness as a control variable we may include in the analysis chronically-ill individuals who report having bad health [19, 20, 58, 59].

Economic status has been characterized by means of four variables, which are also used as control variables. The first is income, on the one hand, and on the other are three indicators that describe social deprivation: the AROPE score (at risk of poverty and social exclusion) and two of its components, severe material deprivation and low work intensity in the household (LWIH).

Income was measured using the equivalent available per capita income of households at constant levels, for the last year of the per capita income series and after logarithmic transformation.

The risk of poverty and social exclusion was measured through the AROPE score. This indicator expands on the traditional relative poverty score by combining it with severe material deprivation and low work intensity in the household. This provides a multidimensional picture of poverty and social exclusion. The AROPE score thus comprises three sub-indicators representing three distinct population sets: individuals at risk of poverty, individuals suffering from severe material deprivation, and individuals living in households characterized by low work intensity. Whenever one such indicator is present, an individual can be safely described as being at risk of poverty or social exclusion [60]. The variable “low work intensity” refers to individuals living in households in which those of working age did work less than 20% of their full potential in the year before the survey. As for severe material deprivation, this describes the share of the population living in households lacking in at least four out of nine specific quality-of-life items. For the third level, which deals with the region, per capita public health expenditure was used. A one-year delay was introduced in order to better reflect any potential link with the perceived health of residents in the region.

All monetary variables, such as income and health expenditure, have been expressed using 2017 prices.

As we are interested in gender differences in educational gradient in health, we have tested models with gender and education as the only explanatory variables (Annex 2 in S1 File). The coefficients are consistent.

After performing an analysis of the degree of multicolinearity between independent variables, it can be safely concluded that they are not an obstacle for the interpretation of results (Annex 3 in S1 File).

## Results

Table 1 shows the frequency of each variable in the model, split by gender.

**Table 1:** Descriptive statistical values, according to gender, of the variables used to measure the relation of educational level and working status with perceived health for Spain between 2014 and 2017.

	Women										Men									
	2014		2015		2016		2017		Total		2014		2015		2016		2017		Total	
	n = 2668	%	n = 4997	%	n = 7104	%	n = 6087	%	n = 20856	%	n = 2573	%	n = 4817	%	n = 6966	%	n = 5899	%	n = 20255	%
**Categorical variables (level 2)**																				
**Perceived health**																				
** Very Good**	526	19.72	934	18.69	1224	17.23	1364	22.41	4049	19.41	544	21.14	1001	20.78	1402	20.13	1426	24.17	4373	21.59
** Good**	1609	60.31	3157	63.18	4561	64.20	3713	61.00	13040	62.52	1622	63.04	3006	62.40	4429	63.58	3593	60.91	12649	62.45
** Fair (neither good nor bad)**	420	15.74	722	14.45	1069	15.05	812	13.34	3022	14.49	313	12.16	640	13.29	931	13.36	710	12.04	2595	12.81
** Bad**	87	3.26	147	2.94	201	2.83	166	2.73	601	2.88	77	2.99	130	2.70	168	2.41	135	2.29	510	2.52
** Very bad**	26	0.97	37	0.74	49	0.69	32	0.53	144	0.69	17	0.66	40	0.83	36	0.52	35	0.59	128	0.63
**Chronic ill-ness**																				
** Yes**	630	23.61	1206	24.13	1641	23.10	1293	21.24	4770	22.87	554	21.53	1150	23.87	1505	21.60	1140	19.33	4349	21.47
** No**	2038	76.39	3791	75.87	5463	76.90	4794	78.76	16086	77.13	2019	78.47	3667	76.13	5461	78.40	4759	80.67	15906	78.53
**Education level**																				
** Primary**	328	12.29	525	10.51	701	9.87	487	8.00	2041	9.79	356	13.84	571	11.86	784	11.25	546	9.26	2256	11.14
** Secondary**	1451	54.39	2750	55.03	3899	54.89	3318	54.51	11419	54.75	1495	58.09	2813	58.39	4091	58.73	3476	58.92	11876	58.63
** College**	889	33.32	1722	34.46	2504	35.24	2282	37.49	7396	35.46	722	28.07	1433	29.75	2091	30.02	1877	31.82	6123	30.23
**Working status**																				
** Employed**	1371	51.37	2652	53.07	3857	54.30	3452	56.70	11332	54.33	1562	60.70	3107	64.50	4490	64.45	3979	67.46	13139	64.87
** Unemployed**	577	21.62	1025	20.50	1367	19.27	960	15.75	3927	18.83	597	23.22	936	19.44	1256	18.03	911	15.45	3701	18.27
** Student**	279	10.46	552	11.04	794	11.20	673	11.06	2296	11.01	269	10.42	497	10.31	813	11.67	644	10.91	2220	10.96
** Homemaker**	340	12.76	600	12.01	820	11.56	735	12.12	2496	11.97	2	0.07	4	0.08	10	0.14	9	0.15	24	0.12
** Retired**	7	0.25	7	0.15	18	0.26	31	0.51	65	0.31	22	0.87	37	0.76	61	0.87	77	1.30	197	0.97
** Other inactive**	94	3.54	161	3.23	248	3.50	236	3.87	740	3.55	121	4.72	236	4.91	336	4.84	279	4.73	974	4.81
**Severe material deprivation**																				
** Yes**	169	6.35	233	4.67	305	4.30	186	3.05	895	4.29	147	5.70	221	4.59	277	3.98	183	3.10	828	4.09
** No**	2499	93.65	4764	95.33	6799	95.70	5901	96.95	19961	95.71	2426	94.30	4596	95.41	6689	96.02	5716	96.90	19427	95.91
**LWIH**																				
** Yes**	558	20.90	855	17.11	1148	16.16	876	14.39	3437	16.48	500	19.44	802	16.65	1059	15.20	763	12.94	3125	15.43
** No**	2110	79.10	4142	82.89	5956	83.84	5211	85.61	17419	83.52	2073	80.56	4015	83.35	5907	84.80	5136	87.06	17130	84.57
**AROPE**																				
** Yes**	940	35.24	1593	31.87	2196	30.91	1789	29.39	6518	31.25	865	33.60	1467	30.46	2027	29.10	1588	26.92	5947	29.36
** No**	1728	64.76	3404	68.13	4908	69.09	4298	70.61	14338	68.75	1708	66.40	3350	69.54	4939	70.90	4311	73.08	14308	70.64
**Continuous variables**	**M**	**SD**	**M**	**SD**	**M**	**SD**	**M**	**SD**	**M**	**SD**	**M**	**SD**	**M**	**SD**	**M**	**SD**	**M**	**SD**	**M**	**SD**
**Age (level 2)**	40.53	12.43	40.61	12.58	41.07	12.68	41.33	12.83	40.96	12.67	39.72	12.50	40.21	12.65	40.41	12.79	40.78	12.93	40.38	12.77
**Household income (level 2)**	9.41	0.74	9.45	0.76	9.49	0.76	9.50	0.75	9.47	0.76	9.44	0.71	9.47	0.74	9.51	0.72	9.53	0.72	9.50	0.72
**Per capita health expenditure (level 3)**	1293.42	150.23	1385.97	146.46	1412.39	159.10	1410.20	156.12	1390.16	158.89	1288.63	149.44	1387.39	145.69	1412.28	159.10	1411.84	156.43	1390.47	159.15

*Note*. LWIH (Low Work Intensity in the Household) AROPE (At Risk of Poverty and Social Exclusion).Source: Prepared by the authors using data from Instituto Nacional de Estadistica (INE) (2020). Carencia Material. Carencia Material Severa [Material Deprivation. Severe Material Deprivation]. Recovered from https://www.ine.es/ss/Satellite?L=es_ES&c=INESe cion_C&cid=1259925456180&p=1254735110672&pagename=ProductosYServicios%2FPYSLayout&param1=PYSDetalle&param3=1259924822888, Instituto Nacional de Estadística (INE) (2022). Encuesta de condiciones de vida. Resultados. Recovered from https://www.ine.es/dyngs/INEbase/es/operacion.htm?c=Estadistica_C&cid=1254736176807&menu=resultados&idp=1254735976608#!tabs-1254736195153 and Ministerio de Sanidad del Gobierno de España (2022) Estadística de Gasto Sanitario Público (EGSP) 2019: Principales resultados. R covered from: https://www.mscbs.gob.es/estadEstudios/estadisticas/docs/EGSP2008/egspPrincipalesResultados.pdf.LWIH (Low Work Intensity in the Household) AROPE (At Risk of Poverty and Social Exclusion)

https://www.ine.es/ss/Satellite?L=es_ES&c=INESeccion_C&cid=1259925456180&p=1254735110672&pagename=ProductosYServicios%2FPYSLayout&param1=PYSDetalle&param3=1259924822888 According to data in Table 1, the share of individuals describing their health as fair, bad, or very bad has increased between 2014 and 2016, and only slightly decreased in 2017. Such temporary respite affected women and men alike. As for the variables of interest, the prevailing educational level for women and men in the whole series under analysis is secondary education, followed by college-level studies. As far as working status is concerned, for the period under analysis we observed a sustained increased in the number of employed individuals, with a corresponding decrease in unemployment rates, also for women as well as men. The working status category displaying the largest gap is that of homemakers, which includes almost 12% of women but only 0.12% of men.

Regarding economic status, both women and men enjoyed higher income levels in the last year under analysis: 2017. The three indicators that describe social deprivation successively decreased over the 2014–2017 period for both genders alike.

To begin with, for the whole set of individuals under analysis we estimated both a multilevel linear model and a logit multilevel model. Table 2 displays the results of these estimations. In order to ease the interpretation of results in the global linear model, we have calculated effect sizes for the variables of interest, as shown in Table 3.

**Table 2:** Random intercept linear and logistic multilevel model for the exploration of associations of individual educational level and working status with perceived health by gender. Spain (2014–2017).

	**Global**									
	**Linear model**					**Logistic model**				
**Variable**	**Coefficient**	**SE**	**95% CI**		**p**	**Odds ratio**	**SE**	**95% CI**		**p**
			*LL*	*UL*				*LL*	*UL*	
**Working status (level 2)**										
** • Employed**	Reference	Reference				Reference	Reference			
** • Unemployed**	.039	.014	.010	.068	.006	1.232	.076	1.089	1.394	.001
** • Student**	-.049	.013	-.076	-.023	.000	.861	.141	.624	1.188	.361
** • Homemaker**	.014	.100	-.005	.034	.149	1.078	.631	.962	1.210	.196
** • Retired**	.140	.055	.031	.248	.012	1.540	.379	.950	2.496	.080
** • Other inactive**	.495	.330	.431	.560	.000	4.100	.492	3.241	5.187	.000
**Educational level (level 2)**										
** • Primary**	.051	.011	.029	.073	.000	1.228	.075	1.090	1.384	.001
** • Secondary**	Reference	Reference				Reference	Reference			
** • College**	-.089	.012	-.114	-.065	.000	.667	.037	.598	.745	.000
**Social deprivation (level 2)**										
** • LWIH**	.048	.017	.016	.081	.004	1.010	.115	.805	1.260	.952
** • Severe material Deprivation**	.144	.024	.096	.192	.000	1.856	.177	1.540	2.234	.000
** • AROPE**	.053	.015	.017	.089	.001	1.437	.105	1.244	1.658	.000
** • Household income (level 2)**	-.020	.007	-.034	-.006	.006	. 912	.037	.843	.988	.023
**Per capita health expenditure (level 3)**	.000	.000	-.000	.000	.347	1.000	.000	1.000	1.001	.242
**Gender (level 2)**										
** • Women**	.042	.008	.023	.062	.000	1.187	.063	1.070	1.318	.001
**Age (level 2)**	.012	.000	.011	.013	.000	1.048	.003	1.042	1.055	.000
**Chronic illness (level 2)**	.736	.014	.709	.764	.000	19.206	.000	15.619	23.618	.000
**Constant**	1.382	.136	1.113	1.651	.000	.007	.006	.011	.039	.000

	**Women**										**Men**									
	**Linear model**					**Logistic model**					**Linear model**					**Logistic model**				
**Variable**	**Coefficient**	**SE**	**95% CI**		**p**	**Odds ratio**	**SE**	**95% CI**		**p**	**Coefficient**	**SE**	**95% CI**		**p**	**Odds ratio**	**SE**	**95% CI**		**p**
			*LL*	*UL*				*LL*	*UL*				*LL*	*UP*				*LL*	*UL*	
**Working status (level 2)**																				
** • Employed**	Reference	Reference				Reference	Reference				Reference	Reference				Reference	Reference			
** • Unemployed**	.013	.026	-.038	.065	.617	1.103	.105	.916	1.328	.302	.069	.016	.037	.102	.000	1.401	.130	1.168	1.680	.000
** • Student**	-.062	.019	-.995	-.024	.001	.773	.137	.547	1.094	.146	-. 034	.014	-.061	-.006	.015	.971	.240	.599	1.574	.906
** • Homemaker**	.001	.012	-.024	.257	.934	1.028	.073	.894	1.182	.700	.045	.157	-.264	.354	.776	1.909	2.560	.138	26.423	.630
** • Retired**	.115	.097	-.074	.305	.234	1.145	.455	.523	2.496	.733	.164	.062	.043	.285	.008	1.793	.531	1.003	3.206	.049
** • Other inactive**	.428	.045	.339	.516	.000	3.224	.460	2.437	4.265	.000	.563	.037	.490	.635	.000	5.136	.643	4.018	6.564	.000
**Educational level (level 2)**																				
** • Primary**	.070	.013	.044	.097	.000	1.314	.102	1.128	1.530	.000	.028	.017	-.004	.061	.090	1.131	.102	.947	1.351	.173
** • Secondary**	Reference	Reference				Reference	Reference				Reference	Reference				Reference	Reference			
** • College**	-.077	.012	-.100	-.053	.000	.702	.045	.619	.797	.000	-.098	.019	-.135	-.061	.000	.620	.047	.535	.719	.000
**Social deprivation (level 2)**																				
** • LWIH**	.053	.025	.004	.103	.034	1.050	.155	.786	1.403	.739	.036	.014	.007	.065	.014	.943	.100	.766	1.160	.579
** • Severe material Deprivation**	.129	.025	.081	.018	.000	1.852	.179	1.534	2.237	.000	.158	.029	.101	.215	.000	1.842	.289	1.355	2.505	.000
** • AROPE**	.046	.024	-.000	.093	.052	1.407	.146	1.148	1.725	.001	.058	.016	.027	.089	.000	1.444	.120	1.223	1.698	.000
** • Household income (level 2)**	-.028	.010	-.049	-.008	.006	.921	.043	.840	1.009	.078	-.007	.015	-.037	.022	.617	.895	.054	.796	1.007	.066
**Per capita health expenditure (level 3)**	.000	.000	-.000	.000	.404	1.000	.000	1.000	1.001	.152	.000	.000	-.000	.000	.208	1.000	.000	1.000	1.001	.294
**Age (level 2)**	.012	.001	.010	.013	.000	1.047	.006	1.036	1.058	.000	.013	.001	.011	.014	.000	1.050	.002	1.046	1.055	.000
**Chronic illness (level 2)**	.766	.014	.738	.794	.000	19.896	2.349	15.786	25.076	.000	.701	.022	.659	.744	.000	18.445	1.898	15.076	22.566	.000
**Constant**	1.527	.123	1.287	1.768	.000	0.008	.006	.002	.036	.000	1.189	.220	.758	1.620	.000	.008	.008	.001	.063	.000

*Note*. N (level 1: years) = 4; N(level 2: women) = 8.603; N(Level 2: men) = 8.424; N(level 3 (regions) = 17. LWIH (Low Work Intensity in the Household) AROPE (At Risk of Poverty and Social Exclusion)

Source: Prepared by the authors using data from Instituto Nacional de Estadistica (INE) (2020). Carencia Material. Carencia Material Severa [Material Deprivation. Severe Material Deprivation]. Recovered from https://www.ine.es/ss/Satellite?L=es_ES&c=INESeccion_C&cid=1259925456180&p=1254735110672&pagename=ProductosYServicios%2FPYSLayout&param1=PYSDetalle&param3=1259924822888, Instituto Nacional de Estadística (INE) (2022). Encuesta de condiciones de vida. Resultados. Recovered from https://www.ine.es/dyngs/INEbase/es/operacion.htm?c=Estadistica_C&cid=1254736176807&menu=resultados&idp=1254735976608#!tabs-1254736195153 and Ministerio de Sanidad del Gobierno de España (2022) Estadística de Gasto Sanitario Público (EGSP) 2019: Principales resultados. Recovered from: https://www.mscbs.gob.es/estadEstudios/estadisticas/docs/EGSP2008/egspPrincipalesResultados.pdf.

**Table 3:** Size of effects for interest independent variables for the exploration of associations of individual educational level and working status with perceived health by gender. Spain (2014–2017).

Independent variables of interest	Global linear model
**Women**	0.106%
**Working status**	
** • Employed**	Reference
** • Unemployed**	0.035%
** • Student**	-0.026%
** • Homemaker**	n.s.
** • Retired**	0.004%
** • Other inactive**	0.103%
**Education level**	
** • Primary**	0.026%
** • Secondary**	Reference
** • College**	-0.146%
**Per capita health expenditure**	n.s.

Note. n.s.: not significant Source: Prepared by the authors using data from Instituto Nacional de Estadística (INE) (2022). Encuesta de condiciones de vida. Resultados. Recovered from https://www.ine.es/dyngs/INEbase/es/operacion.htm?c=Estadistica_C&cid=1254736176807&menu=resultados&idp=1254735976608#!tabs-1254736195153 and Ministerio de Sanidad del Gobierno de España (2022) Estadística de Gasto Sanitario Público (EGSP) 2019: Principales resultados. Recovered from: https://www.mscbs.gob.es/estadEstudios/estadisticas/docs/EGSP2008/egspPrincipalesResultados.pdf

From the results displayed in Table 3, being a woman is associated with 0.106% poorer self-rated health compared with men, this being the most harmful variable among all included. In contrast, having received a college-level education displays the strongest relation to reporting improved health, 0.146% over individuals who have a secondary education level.

The results in Table 2 are consistent across both models for the variables of interest: unemployment and educational attainment level.

As regards educational level, having successfully completed only a primary-level education is associated with reporting worse self-perceived health (β = 0.051, p = 0.000; OR = 1.228, p = 0.001), whereas having received a college-level education is associated with reporting better health (β = − 0.089, p = 0.000; OR = 0.667, p = 0.000), in contrast with having received a secondary education.

As for working status, being unemployed is associated with reporting worse health than employed individuals (β = 0.039, p = 0.006; OR = 1.232, p = 0.001).

As a variable, gender is significantly associated with an individual’s health. Specifically, being a woman is associated with reporting poorer health than men do (β = 0.042, p = 0.000; odds ratio = 1.187, p = 0.001).

In order to delve deeper into any possible asymmetries regarding gender and its association with the socio-economic variables of interest, separate analyses were conducted for men and women. Table 2 displays the results separately, with two specifications for each model: linear and logit.

In matters of education, having successfully completed only a primary-level education is associated with reporting poor self-perceived health in the case of women (β = 0.070, p = 0.00; OR = 1.314, p = 0.000), whereas having received a college-level education is associated with reporting better health (β = − 0.077, p = 0.00; OR = 0.702, p = 0.000), in contrast with being in possession of a secondary education. Conversely, for men a primary education level was not significant for their health (β = 0.028, p = 0.090; OR = 1.131, p = 0.173) but having undergone higher education is associated with reporting better health (β = -0.098, p = 0.000; OR = 0.620, p = 0.000) than individuals who only have attained a secondary education level.

As for working status, the perceived health of Spanish women does not appear to be different for the unemployed in comparison with those under employment (β = 0.013, p = 0.617; OR = 1.103, p = 0.302), whereas for men unemployment is associated with reporting one’s health as poorer than that of employed individuals. Unemployed men were, on average, 0.07 points below their employed peers in perceived health (the logit model yielded similar results, OR = 1.401, p = 0.000). Similarly, being retired is not associated with bad health in the case of employed women (β = 0.115, p = 0.234; OR = 1.145, p = 0.733), while for men it worsens their self-perceived health (β = 0.164, p = 0.000; OR = 1.793, p = 0.049).

Income is a protective factor (β = − 0.028, p < 0.05), whereas severe material deprivation (β = 0.129, p = 0.00), living in a low work intensity household (β = 0.053, p < 0.05), and the AROPE rating (β = 0.046, p < 0.10) are definite risk factors. Finally, as regards working status, only belonging to the other inactive (β = 0.428, p = 0.00) or student categories (β = − 0.062, p < 0.05) yielded statistically significant results. It is worth pointing out that all other working status categories lacked statistical significance, notably being unemployed (β = 0.013, p = 0.617) and being a homemaker (β = 0.001, p = 0.934). The per capita public health expenditure variable was not statistically significant (β = 0.000, p = 0.404) and therefore is not associated with better or worse perceived health among women. The analysis of the economic status of men yielded the following results: income did not reach statistical significance (β = − 0.007, p = 0.617), whereas severe material deprivation (β = 0.158, p = 0.000), living in a household with low work intensity (β = 0.036, p < 0.05), and the AROPE score (β = 0.058, p = 0.000) appeared as risk factors. Finally, and concerning working status, being unemployed (β = 0.069, p = 0.000) and retired (β = 0.164, p < 0.05) were definite risk factors for men. In matters of educational level, for women having received only a primary education worsened their perceived health by 0.07 points (in contrast with being in possession of a secondary education). Conversely, for men having only received a primary education was not a risk factor for health. As for the control variables, it may be stated that those describing economic status, be they severe material deprivation, LWIH, or the AROPE score, are associated with worse health for both women and men.

To sum up, as far as the analysis of gender differences is concerned, for women having a low educational level is associated with reporting worse health, whereas being retired and unemployed are negatively associated with the health of men and men alone. Regional per capita public health expenditure is not statistically associated with the perceived health of women nor men.

## Discussion

The goal of this analysis was to provide a gender perspective on the association of perceived health with educational level and working status among the Spanish population between the years of 2014 and 2017.

This multilevel analysis employed data from the Living Conditions Survey and from several regional sources, and its results suggest that, although certain characteristics are associated with poor reported health among both women and men, others have a differential effect. This points at strong gender differences as far as health and its socio-economic determinants are concerned.

Firstly, our results show that women with low educational attainment levels report their health as being poorer than that of other, more highly-educated women, but the same effect is not apparent among men. In agreement with previous studies [19, 20, 22, 24, 29, 32, 47], we found that women’s health is further improved by the intrinsic rewards of education, as they have fewer other resources from which they can draw in the absence of a degree, as posited by the theory of resource substitution [24]. Whenever women are able to complete an education, their health is improved, often at a higher rate than men’s. This illustrates the point that women are especially reliant on their own education in order to thrive, and therefore improvements in education may reduce their health-damaging behaviors further than those of men [25].

This result suggests that the meticulous design of educational policies may indirectly reduce the gender-based health gap, particularly among women of a lower educational level [61].

On the one hand, the guarantee of a universal primary education aimed at ensuring that all girls attend school definitely translates to an improvement in the health of women, as revealed by the available evidence. Heymann et al. (2019) analyzed the effectiveness of educational policies in improving gender equality and its related health outcomes in the 193 countries of the UN, and identified primary education policies and regulations as major contributors to the health of women and girls [62]. A 10% rise in educational parity, which only amounts to a 4.9% increase in years of schooling for girls, was associated with a 2.06 increase in life expectancy for women and a 0.88 increase in male life expectancy at birth.

On the other hand, further health improvements are derived from policies aimed at increasing the active participation in society of women over 50 with low educational achievement levels (including membership in community associations, volunteer work, and caregiving). According to the work of Arpino and Solé-Auró (2019) regarding European individuals over 50, active aging activities are able to explain 12% to 33% of health-related differences between women of a low and high educational attainment levels, whereas for men the range was 16% to 21% [63].

With respect to working status, unemployment is not associated with the perceived health of women, but it worsens that of men, as previously reported by Calzón et al. (2017) [20]. This suggests that the negative association between unemployment and welfare is not as strong for women as it is for men [31]. Gender differences in the deleterious effects of unemployment or subemployment may therefore arise from the fact that men receive more social approval than women by virtue of being employed [64]. The myth of the nuclear family and the concept of manhood as a function of a man’s ability to provide for their family still lie behind currently prevailing notions of masculinity and patriarchy [42, 65]. These misconceptions have failed to adapt to new landscapes of family and work life, which may lead to increased physical and mental health problems for men in times of economic turmoil and uncertainty [64]. As reported by the World Bank (2011), men see access to the labor market as their only avenue for empowerment, and any setback in that regard makes them vulnerable [41].

To conclude, unemployment has a stronger effect on the health of men than on that of women, as it has deleterious effects on their individual and social identity, particularly in countries where traditional and highly-differentiated gender roles endure [45, 66, 67]. In the future, policy development should identify men as a vulnerable population with specific mental health needs [68], and establish community awareness and prevention campaigns aimed at challenging traditional notions of masculinity and redefining male vulnerability to mental health problems [69, 70].

By instituting active labor market programs (ALMPs), the negative health outcomes associated with unemployment may be effectively addressed, alleviating the adverse health effects brought about by economic recessions [11, 71]. To give an example, Reeves et al. (2015) found that increasing ALMP expenditure reduce suicide risks [72], and Mattei et al. 2019 concluded that ALMPs are effective in softening the association between unemployment and suicide among men, in particular between the ages of 45 to 54 [73].

Given that men are often reticent to seek help for their depression, ALMPs that include specific mental health content would be able to make individuals aware of the link between depression and work-related suicide [69]. Similarly, Dillon and Butler (2011) suggested that depression- and suicide-prevention programs should target unemployed men specifically, as they should be considered a high-risk population [66]. Additionally, Okada et al. (2020) concluded that the regional suicide-prevention programs (Emergency Fund to Enhance Community-Based Suicide Countermeasures-EFECBSC) instituted in Japan helped reduce mortality by suicide among unemployed males [74].

Lastly, observations of per capita public health expenditure failed to reveal any association with the perceived health of either women or men. While it is widely understood that it should have a strong effect on the health status of individuals, in developed countries additional expenditure is often unrelated to improvements in perceived health, as previously described in the literature [46–48]. This does not discount the fact that public policy makers should always be on the lookout for new opportunities to improve health outcomes through regionally-distributed health expenditure and efficient policies.

Furthermore, statistics on health outcomes may be improved by a reassignment of resources from healthcare to social programs. This is particularly the case of policies with a strong effect on the social determinants analyzed, such as those of an educational nature. Education is able to create human capital and promote the pre-distribution of income and wealth, remaining to this day the variable with the strongest explanatory power regarding health status and gender differences.

This study has several limitations. To begin with, a causal relation may not be established between the variables under analysis, as this would require the application of other methodologies, more similar to a quasi-experimental study design. However, the large size of the database employed, its longitudinal character, and the robustness of the results concerning several different specifications of the perceived health variable provide a solid starting point for further studies, which may be able to determine in more detail whether the associations found between education and unemployment have a causal nature. Secondly, although the variation partition coefficient is not high, it would be fitting to explore other public policy variables at the regional level, such as social expenditure or unemployment rates. Finally, expanding the time range with data generated after 2017 would enable researchers to assess the long-term stability of the associations found in the present study.

To conclude, policies aimed specifically at improving the educational level of women may contribute to the general health of the population. However, regional public health expenditure does not seem to be associated with better health outcomes, which puts into question the usefulness of general increases in that budgetary chapter.

These results are particularly relevant in the face of the current post-COVID crisis, as they may help guide public policy in matters of education and labor policy with the goal of promoting the post-pandemic recovery of our societies.

## Supporting information

S1 File (DOCX)
