# Supplementary material for: Variation in perceived health across gender, working status, educational level, and regional health care expenditure in Spain (2014–2017)
Source: PLoS One. 2023 Jul 14;18(7):e0269613. doi: 10.1371/journal.pone.0269613 (PMC10348579; doi:10.1371/journal.pone.0269613)
Supplement: S1 File — (DOCX) [file pone.0269613.s001.docx]

**Annex 1:**

*Sintax in Stata 15 for random intercept linear and logistic multilevel models for the exploration of individual educational level and working status with perceive health by gender. Spain (2014-2017).*

*MEN

xtset idpersona año

* LINEAR MODEL
sum salud edad estudios_primaria estudios_superiores si_cronica desempleado estudiante ama_amo_casa jubilados resto_inactivos carenc_mat_sev vrlowjob vreu2020 ln_deflact_rentaequiv gastosanpc

mixed salud edad estudios_primaria estudios_superiores desempleado estudiante ama_amo_casa jubilados resto_inactivos carenc_mat_sev vrlowjob vreu2020 ln_deflact_rentaequiv gastosanpc [pweight = peso_personal], || region:, covariance(unstructured) || idpersona:, covariance(unstructured)

estat icc

* LOGISTIC MODEL
sum mala_salud edad estudios_primaria estudios_superiores si_cronica desempleado estudiante ama_amo_casa jubilados resto_inactivos carenc_mat_sev vrlowjob vreu2020 ln_deflact_rentaequiv gastosanpc

melogit mala_salud edad estudios_primaria estudios_superiores si_cronica desempleado estudiante ama_amo_casa jubilados resto_inactivos carenc_mat_sev vrlowjob vreu2020 ln_deflact_rentaequiv gastosanpc [pweight = peso_personal], || region:, covariance(unstructured) || idpersona:, covariance(unstructured) or difficult iterate(3000)

estat icc

*WOMEN

xtset idpersona año

* LINEAR MODEL
sum salud edad estudios_primaria estudios_superiores si_cronica desempleado estudiante ama_amo_casa jubilados resto_inactivos carenc_mat_sev vrlowjob vreu2020 ln_deflact_rentaequiv gastosanpc

mixed salud edad estudios_primaria estudios_superiores si_cronica desempleado estudiante ama_amo_casa jubilados resto_inactivos carenc_mat_sev vrlowjob vreu2020 ln_deflact_rentaequiv gastosanpc [pweight =peso_personal], || region:, covariance(unstructured) || idpersona:, covariance(unstructured)

estat icc

* LOGISTIC MODEL
sum mala_salud edad estudios_primaria estudios_superiores si_cronica desempleado estudiante ama_amo_casa jubilados resto_inactivos carenc_mat_sev vrlowjob vreu2020 ln_deflact_rentaequiv gastosanpc

melogit mala_salud edad estudios_primaria estudios_superiores si_cronica desempleado estudiante ama_amo_casa jubilados resto_inactivos carenc_mat_sev vrlowjob vreu2020 ln_deflact_rentaequiv gastosanpc [pweight = peso_personal], || region:, covariance(unstructured) || idpersona:, covariance(unstructured) or difficult iterate(1000)

estat icc

*GLOBAL

xtset idpersona año

* LINEAR MODEL
sum salud edad estudios_primaria estudios_superiores si_cronica desempleado estudiante ama_amo_casa jubilados resto_inactivos carenc_mat_sev vrlowjob vreu2020 ln_deflact_rentaequiv gastosanpc

mixed salud edad estudios_primaria estudios_superiores si_cronica desempleado estudiante ama_amo_casa jubilados resto_inactivos mujer carenc_mat_sev vrlowjob vreu2020 ln_deflact_rentaequiv gastosanpc [pweight = peso_personal], || region:, covariance(unstructured) || idpersona:, covariance(unstructured) difficult iterate(3000)

estat icc

* LOGISTIC MODEL
sum mala_salud edad estudios_primaria estudios_superiores si_cronica desempleado estudiante ama_amo_casa jubilados resto_inactivos mujer carenc_mat_sev vrlowjob vreu2020 ln_deflact_rentaequiv gastosanpc

melogit mala_salud edad estudios_primaria estudios_superiores si_cronica desempleado estudiante ama_amo_casa jubilados resto_inactivos mujer carenc_mat_sev vrlowjob vreu2020 ln_deflact_rentaequiv gastosanpc [pweight = peso_personal], || region:, covariance(unstructured) || idpersona:, covariance(unstructured) or difficult iterate(3000)

estat icc

**Annex 2**

*Random intercept linear and logistic multilevel model for the exploration of associations of individual educational level and working status with perceived health by gender. Spain (2014-2017)*

|  | **Lineal model** | | | | | **Logistic model** | | | | | **Lineal model** | | | | | **Logistic model** | | | | |
| --- | --- | --- | --- | --- | --- | --- | --- | --- | --- | --- | --- | --- | --- | --- | --- | --- | --- | --- | --- | --- |
| Variable | **Coefficient** | **SE** | **95% CI** | | **p** | **Odd ratio** | **SE** | **95% CI** | | **P** | **Coefficient** | **SE** | **95% CI** | | **p** | **Odd ratio** | **SE** | **95% CI** | | **p** |
|  |  |  | *LL* | *UL* |  |  |  | *LL* | *UL* |  |  |  | *LL* | *UL* |  |  |  | *LL* | *UL* |  |
| Gender (level 2)   - Women | .055 | .009 | .038 | .073 | .000 | 1.200 | .079 | 1.054 | 1.367 | .006 | .072 | .014 | .044 | .100 | .000 | 1.313 | .098 | .648 | 1.023 | .000 |
| Age (level 2) | - | - |  |  | - | - | - |  |  | - | - | - |  |  | - | - | - |  |  | - |
| Chronic illness | - | - |  |  | - | - | - |  |  | - | - | - |  |  | - | - | - |  |  | - |
| Working status |  |  |  |  |  |  |  |  |  |  |  |  |  |  |  |  |  |  |  |  |
| Employed  Unemployed  Student  Homemaker  Retired | Reference | Reference |  |  |  | Reference | Reference |  |  |  | Reference | Reference |  |  |  | Reference | Reference |  |  |  |
|  | .112 | .018 | .075 | .148 | .000 | 1.594 | .111 | 1.390 | 1.829 | .000 | .134 | .026 | .082 | .186 | .000 | 1.771 | .152 | 1.497 | 2.097 | .000 |
|  | -.353 | .022 | -.395 | -.310 | .000 | .262 | .034 | .203 | .338 | .000 | -.343 | .033 | -.407 | -.278 | .000 | .279 | .074 | .166 | .468 | .000 |
|  | .131 | .015 | .102 | .161 | .000 | 1.557 | .061 | 1.442 | 1.682 | .000 | -.030 | .217 | -.456 | .395 | .000 | 1.476 | 1.303 | .261 | 8.33 | .000 |
|  | .570 | .062 | .447 | .692 | .000 | 5.111 | .996 | 3.488 | 7.488 | .000 | .575 | .085 | .408 | .743 | .000 | 5.557 | 1.411 | 3.378 | 9.139 | .000 |
| Other inactive | .950 | .050 | .851 | 1.049 | .000 | 11.690 | 1.252 | 9.476 | 14.421 | .000 | 1.041 | .050 | .943 | 1.140 | .000 | 15.868 | 2.061 | 12.303 | 20.478 | .000 |
| Education level   - Primary - Secondary - College |  |  |  |  |  |  |  |  |  |  |  |  |  |  |  |  |  |  |  |  |
|  | .175 | .025 | .126 | .224 | .000 | 1.683 | .186 | 1.354 | 2.091 | .000 | .136 | .033 | .072 | .200 | .000 | 1.521 | .204 | 1.170 | 1.978 | .002 |
|  | Reference | Reference |  |  |  | Reference | Reference |  |  |  | Reference | Reference |  |  |  | Reference | Reference |  |  |  |
|  | -.138 | .013 | -.164 | -.112 | .000 | .578 | .021 | .538 | .622 | .000 | -.132 | .022 | -.175 | -.089 | .000 | .575 | .039 | .503 | .656 | .000 |
| Social deprivation   - LWIH - Severe material deprivation - AROPE | -  -  - | -  -  - |  |  | -  -  - | -  -  - | -  -  - |  |  | -  -  - | -  - | -  - |  |  | -  - | -  - | -  - |  |  | -  - |
| Household income | - | - |  |  | - | - | - |  |  | - | - | - |  |  | - | - | - |  |  | - |
| Per capita health expenditure (level 3) | - | - |  |  | - | - | - |  |  | - | - | - |  |  | - | - | - |  |  | - |
| Women X Unemployed | - | - |  |  | - | - | - |  |  | - | -.043 | .047 | -.135 | .048 | .353 | .814 | .095 | .648 | 1.023 | .078 |
| Women X Student | - | - |  |  | - | - | - |  |  | - | -.023 | .032 | -.086 | .040 | .476 | .881 | .272 | .481 | 1.614 | .681 |
| Women X Homemaker | - | - |  |  | - | - | - |  |  | - | .145 | .227 | -.301 | .590 | .524 | .978 | .892 | .164 | 5.839 | .981 |
| Women X Retired | - | - |  |  | - | - | - |  |  | - | .006 | .141 | -.272 | .283 | .966 | .812 | .346 | .353 | 1.872 | .626 |
| Women X Other inactive | - | - |  |  | - | - | - |  |  | - | -.207 | .053 | -.311 | -.104 | .000 | .509 | .048 | .423 | .613 | .000 |
| Women x Primary education | - | - |  |  | - | - | - |  |  | - | .081 | .031 | .019 | .142 | .011 | 1.206 | .151 | .944 | 1.541 | .134 |
| Women X College education | - | - |  |  | - | - | - |  |  | - | -.013 | .026 | -.064 | .039 | .623 | 1.006 | .114 | .805 | 1.257 | .959 |
| Constant | 1.952 | .019 |  |  | .000 | .154 | .160 |  |  | .000 | 1.945 | .021 | 1.902 | 1.987 | .000 | .148 | .017 | .118 | .186 | .000 |

*Note*. N (level 1: years) =4; N(level 2: women) =8.603; N(Level 2: men)=8.424; N(level 3 (regions)= 17. LWIH (Low Work Intensity in the Household) AROPE (At Risk of Poverty and Social Exclusion) Source: Prepared by the authors using data from Instituto Nacional de Estadistica (INE) (2020). Carencia Material. Carencia Material Severa [Material Deprivation. Severe Material Deprivation]. Recovered from https://www.ine.es/ss/Satellite?L=es_ES&c=INESeccion_C&cid=1259925456180&p=1254735110672&pagename=ProductosYServicios%2FPYSLayout&param1=PYSDetalle&param3=1259924822888, Instituto Nacional de Estadística (INE) (2022). Encuesta de condiciones de vida. Resultados. Recovered from https://www.ine.es/dyngs/INEbase/es/operacion.htm?c=Estadistica_C&cid=1254736176807&menu=resultados&idp=1254735976608#!tabs-1254736195153 and Ministerio de Sanidad del Gobierno de España (2022) Estadística de Gasto Sanitario Público (EGSP) 2019: Principales resultados. Recovered from: <https://www.mscbs.gob.es/estadEstudios/estadisticas/docs/EGSP2008/egspPrincipalesResultados.pdf>

**Annex 2**

*Random intercept linear and logistic multilevel model for the exploration of associations of individual educational level and working status with perceived health by gender. Spain (2014-2017)*

|  | **Lineal model** | | | | | **Logistic model** | | | | |
| --- | --- | --- | --- | --- | --- | --- | --- | --- | --- | --- |
| Variable | **Coefficient** | **SE** | **95% CI** | | **p** | **Odd ratio** | **SE** | **95% CI** | | **P** |
|  |  |  | *LL* | *UL* |  |  |  | *LL* | *UL* |  |
| Gender (level 2)   - Women | .046 | .010 | .027 | .065 | .000 | 1.228 | .078 | 1.084 | 1.391 | .001 |
| Age (level 2) | .012 | .000 | .011 | .013 | .000 | 1.049 | .003 | 1.042 | 1.055 | .000 |
| Chronic illness | .736 | .014 | .709 | .764 | .000 | 19.235 | 2.048 | 15.613 | 23.698 | .000 |
| Working status   - Employed - Unemployed - Student - Homemaker - Retired - Other inactive |  |  |  |  |  |  |  |  |  |  |
|  | Reference | Reference |  |  |  | Reference | Reference |  |  |  |
|  | .067 | .017 | .034 | .100 | .000 | 1.388 | .108 | 1.191 | 1.618 | .000 |
|  | -.043 | .190 | -.080 | -.006 | .024 | .935 | .248 | 0.556 | 1.574 | .802 |
|  | .037 | .162 | -.280 | .354 | .819 | 1.906 | 2.559 | .137 | 23.486 | .631 |
|  | .161 | .064 | .035 | .287 | .012 | 1.774 | .553 | .964 | 3.268 | .066 |
|  | .548 | .036 | .478 | .619 | .000 | 4.976 | .619 | 3.900 | 6.350 | .000 |
| Education level   - Primary - Secondary - College |  |  |  |  |  |  |  |  |  |  |
|  | .030 | .015 | .001 | .059 | .044 | 1.140 | .100 | .960 | 1.356 | .136 |
|  | Reference | Reference |  |  |  | Reference | Reference |  |  |  |
|  | -.095 | .018 | -.131 | -.060 | .000 | .615 | .046 | .532 | .711 | .000 |
| Social deprivation   - LWIH - Severe material deprivation - AROPE | .009  .143  .069 | .011  .023  .015 | -.013  .098  .040 | .030  .189  .098 | .436  .000  .000 | .981  1.849  1.439 | .073  .169  .089 | .848  1.545  1.275 | 1.135  2.213  1.624 | .797  .000  .000 |
| Household income | -.018 | .007 | -.033 | -.004 | .014 | .910 | .037 | .841 | .985 | .020 |
| Per capita health expenditure (level 3) | .000 | .000 | -.000 | .000 | .713 | 1.000 | .000 | 1.000 | 1.001 | .226 |
| Women X Unemployed | -.037 | .036 | -.107 | .033 | .303 | .806 | .096 | .637 | 1.019 | .071 |
| Women X Student | -.011 | .019 | -.048 | .027 | .567 | .860 | .224 | .517 | 1.432 | .563 |
| Women X Homemaker | -.027 | .165 | -.351 | .297 | .871 | .540 | .731 | .038 | 7.668 | .649 |
| Women X Retired | -.028 | .120 | -.265 | .208 | .813 | .653 | .364 | .219 | 1.948 | .445 |
| Women X Other inactive | -.089 | .041 | -.0171 | -.009 | .031 | .666 | .059 | .559 | .792 | .000 |
| Women x Primary education | .046 | .014 | .018 | .074 | .001 | 1.145 | .134 | .910 | 1.441 | .249 |
| Women X College education | .012 | .017 | -.021 | .045 | .483 | 1.153 | .099 | .974 | 1.366 | .098 |
| Constant | 1.413 | .155 | 1.109 | 1.717 | .000 | .007 | .006 | .001 | .037 | .000 |

*Note*. N (level 1: years) =4; N(level 2: women) =8.603; N(Level 2: men)=8.424; N(level 3 (regions)= 17. LWIH (Low Work Intensity in the Household) AROPE (At Risk of Poverty and Social Exclusion) Source: Prepared by the authors using data from Instituto Nacional de Estadistica (INE) (2020). Carencia Material. Carencia Material Severa [Material Deprivation. Severe Material Deprivation]. Recovered from https://www.ine.es/ss/Satellite?L=es_ES&c=INESeccion_C&cid=1259925456180&p=1254735110672&pagename=ProductosYServicios%2FPYSLayout&param1=PYSDetalle&param3=1259924822888, Instituto Nacional de Estadística (INE) (2022). Encuesta de condiciones de vida. Resultados. Recovered from https://www.ine.es/dyngs/INEbase/es/operacion.htm?c=Estadistica_C&cid=1254736176807&menu=resultados&idp=1254735976608#!tabs-1254736195153 and Ministerio de Sanidad del Gobierno de España (2022) Estadística de Gasto Sanitario Público (EGSP) 2019: Principales resultados. Recovered from: <https://www.mscbs.gob.es/estadEstudios/estadisticas/docs/EGSP2008/egspPrincipalesResultados.pdf>

Annex 3: Analysis of the degree of multicolinearity between independent variables for the exploration of associations of individual educational level and working status with perceived health by gender. Spain (2014-2017)

In order to analyze whether linear relations between independent variables (multicolinearity) are having an effect on our results, it is often advantageous to specify which distinctions are being made. By way of illustration, in Marquardt and Snee (1975) essential multicolinearity designates the linear relation between independent variables after the constant term has been excluded. Contrastingly, non-essential colinearity is the linear relation between the constant term and the rest of the independent variables.

This is an interesting distinction to make, as not all tools employed in determining whether we must worry about approximate multicolinearity are able to detect both types.

In that regard, Salmerón et al. (2020) established that Variance Inflation Factor (VIF) is only able to detect the essential type and suggests using Coefficient of Variation (CV) to detect the non-essential one.

As for the specific values, VIF values over 10 (see, for instance, Salmerón et al. (2018)) and CV values under 0.1 (see Salmerón et al. (2020)) point, respectively, to worrying essential and noon essential colinearity.

As for the values yielded by our analysis, we may confidently state that the degree of essential multicolinearity falls well beneath any concerning threshold, whereas the variable of the Napierian logarithm of income is linked to the constant term.

We face therefore, a problem linked to non-essential multicolinearity due to the relation between the constant term and the logarithm of income (keeping in mind that expenditure, while over 0.1, sits very near the latter). This could be simply solved by focusing on the culprit variable, although such approach would overly complicate the interpretation of its estimated coefficient.

However, as illustrated by Salmerón et al. (2019) (Table 2), this type of multicolinearity only affects the estimation and inference of the constant term, which is why result on the logarithm of income are stable and can be interpreted without further complication.

In summary, the concerning degree of multicolinearity detected would not affect in any case the interpretation of results.

References in order of appearance:

- Marquardt y Snee (1975): [Document Zbl 0361.62060 - zbMATH Open](https://zbmath.org/0361.62060).
- Salmerón et al. (2020): [Diagnosis and quantification of the non-essential collinearity | SpringerLink](https://link.springer.com/article/10.1007/s00180-019-00922-x).
- Salmerón et al. (2018): [Variance Inflation Factor and Condition Number in multiple linear regression: Journal of Statistical Computation and Simulation: Vol 88, No 12 (tandfonline.com)](https://www.tandfonline.com/doi/abs/10.1080/00949655.2018.1463376?journalCode=gscs20).
- Salmerón et al. (2019): [Comment on “A Note on Collinearity Diagnostics and Centering” by Velilla (2018): The American Statistician: Vol 74, No 1 (tandfonline.com)](https://www.tandfonline.com/doi/abs/10.1080/00031305.2019.1635527?journalCode=utas20).
